# Synergistic therapy for diabetic retinopathy via dual blockade of VEGF-A and TNF-α with a bispecific nanobody

**DOI:** 10.3389/fphar.2025.1729606

**Published:** 2026-01-06

**Authors:** Chenlu Wu, Yue Shen, Chengnan Xu, Chong Bi, Chenyue Yan, Jinping Niu, Wenyun Zheng, Xingyuan Ma

**Affiliations:** 1 School of Biotechnology and State Key Laboratory of Bioreactor Engineering, East China University of Science and Technology, Shanghai, China; 2 School of Pharmacy, Shanghai Key Laboratory of New Drug Design, East China University of Science and Technology, Shanghai, China

**Keywords:** bispecific nanobody, diabetic retinopathy, dual-targeting, TNF-α, VEGF-A

## Abstract

**Introduction:**

Anti-vascular endothelial growth factor (VEGF) therapy fails in approximately 30% of diabetic retinopathy (DR) patients, indicating the limitations of solely targeting VEGF-A. This study aimed to design a bispecific nanobody, Nb-TV, targeting both tumor necrosis factor-α (TNF-α) and VEGF-A to achieve dual anti-inflammatory and anti-angiogenic effects for potential DR treatment.

**Methods:**

The inhibitory effects of Nb-TV were evaluated *in vitro* using adult retinal pigment epithelium-19 (ARPE-19) cells and human umbilical vein endothelial cells (HUVECs). Key assays included analysis of nuclear translocation (p65, p38α), phosphorylation (Erk1/2), expression of inflammatory factors (IL-6, IL-8, MCP-1) and inducible nitric oxide synthase (iNOS), Caspase-1 activation, and assessments of HUVEC proliferation, migration, and tube formation. The *in vivo* therapeutic efficacy was investigated in a DR mouse model, where Nb-TV was administered via eye drops. Retinal levels of TNF-α and VEGF-A, neovascularization, and structural lesions were examined.

**Results:**

*In vitro*, Nb-TV inhibited over 60% of TNF-α-induced p65 nuclear translocation in ARPE-19 cells, significantly reducing IL-6, IL-8, and MCP-1 expression by 24.6%, 26.6%, and 37.8%, respectively, and suppressing Caspase-1 activation. In HUVECs, it effectively blocked VEGF-A-driven proliferation, migration, and tube formation by inhibiting p38α nuclear translocation (28.5% reduction) and Erk1/2 phosphorylation (98.6% inhibition), while downregulating iNOS expression. *In vivo*, topical Nb-TV application decreased TNF-α and VEGF-A levels in retinal tissues of DR mice, controlled pathological neovascularization, and improved retinal structural damage.

**Discussion:**

Our findings demonstrate that Nb-TV mechanistically dual-targets the TNF-α/NF-κB and VEGF-A/MAPK pathways, mitigating both inflammation and angiogenesis synergistically. This validates Nb-TV as a novel bispecific nanobody with promising therapeutic potential for DR by simultaneously modulating key pathological pathways. This study not only proposes a strategic approach for refractory DR but also provides a framework for targeting other multifactorial ocular and systemic diseases.

## Graphical Abstract

**Figure:**
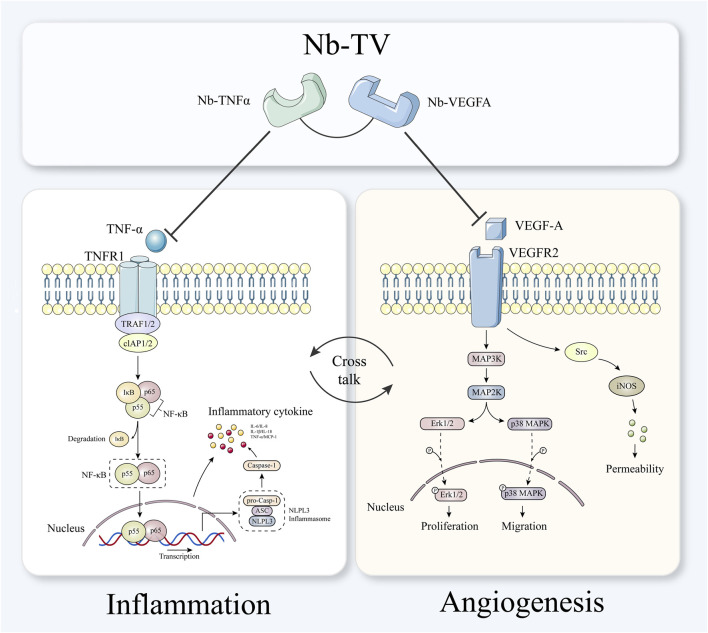

## 1 Introduction

Diabetic retinopathy, which represents a progressive microvascular complication associated with diabetes mellitus (DM), is widely recognized as the principal cause of preventable blindness among both the working-age and elderly cohorts (Antonetti et al., 2012). As per the projection formulated by the American Academy of Ophthalmology in 2021, the global burden of DR is projected to remain elevated over the subsequent 2 decades, with the estimated number of DR patients worldwide expected to reach 160 million by 2045 (Teo et al., 2021).

Since 1948, the pro-inflammatory mechanisms in DR have attracted extensive attention (Rodriguez and Root, 1948). Research findings have demonstrated that elevated levels of pro-inflammatory cytokines, such as TNF-α, nitric oxide (NO), insulin-like growth factor-1 (IGF-1), and IL-6, have been detected in the vitreous humor and retina of both early-stage patients with diabetes and animal models (Capitão and Soares, 2016). Retinal neovascularization is the main characteristic of the progression of diabetic retinopathy. Accordingly, diabetic retinopathy can be categorized into two distinct forms: non-proliferative diabetic retinopathy (NPDR) and proliferative diabetic retinopathy (PDR) (Wilkinson et al., 2003). Neovascularization can lead to visual impairment, intraocular hemorrhage and metabolite leakage. The elevated levels of pro-angiogenic factors such as VEGF-A, angiopoietin-2 (Ang-2) and IL-8 are the key factors in angiogenesis (Rodríguez et al., 2019). In addition, crosstalk occurs between inflammation and angiogenesis. Inflammatory cells can generate cytokines that contribute to the formation of new blood vessels, and pro-angiogenic factors also participate in the recruitment of leukocytes and the secretion of pro-inflammatory factors (Heloterä and Kaarniranta, 2022).

TNF-α exerts a crucial role in inflammation, apoptosis and the development of the immune system. It engages in the body’s inflammatory and immune responses (Yu et al., 2020) via activating the NF-κB signaling pathway and synchronously modulates the generation of downstream cytokines, cell survival and death, thus sustaining the body’s homeostasis. Müller cells are macroglial cells within the retina. Under high-glucose circumstances, Müller cells secrete TNF-α and exert an effect on retinal pigment epithelium (RPE) cells (Portillo et al., 2017). Subsequent to the activation of the NF-κB pathway, RPE cells secrete copious amounts of inflammatory factors and chemokines, including IL-6 (Robinson et al., 2022), IL-8 (Feng et al., 2018) and MCP-1 (Dong et al., 2018), thereby leading to a vicious cycle of ocular inflammatory damage and inflammatory response.

VEGF plays an essential role in the growth, migration, and survival of endothelial cells. In the human retina, VEGF-A is predominantly generated by RPE cells, astrocytes, Müller cells, endothelial cells, and ganglion cells (Arrigo et al., 2022). In the context of DR, ischemia, oxidative stress, and inflammation will potently stimulate the release of VEGF-A (Antonetti et al., 1999). The binding of VEGF-A to VEGFR will mediate angiogenesis-related pathways, such as p38 MAPK, PLCγ/Erk1/2, PI3K/Akt (Simons et al., 2016), *etc*., and mediate endothelial cell survival, vascular permeability, and migratory/proliferative phenotypes (Kerbel, 2008).

The treatment of PDR is arduous. Given the significant role of inflammation in DR, corticosteroids, which directly or indirectly counteract the release of cytokines and chemokines, are regarded as effective anti-inflammatory agents. The majority of studies on corticosteroid treatment for DR concentrate on triamcinolone acetonide, dexamethasone, and fluocinolone (Semeraro et al., 2019). Corticosteroids alleviate retinal inflammation; however, their application is restricted due to potential side effects like cataracts and glaucoma. Targeted inhibition of VEGF is a prominent research focus in the treatment of DR. Anti-VEGF agents including bevacizumab, ranibizumab, aflibercept and faricimab have demonstrated advantages in terms of visual acuity improvement. Although anti-VEGF therapy has achieved effective progress and is currently the preferred treatment for DR, 40% of cases remain refractory and poorly responsive (Tomita et al., 2021). DR involves multiple aspects, including inflammation, angiogenesis, and neurodegeneration. Identifying specific targets within this complex interaction network is challenging. Single-target therapy has limitations, and a multi-target approach offers novel and promising alternatives for the treatment of exudative retinal diseases.

Nanobody (with a molecular weight of approximately 15 kDa), consisting of only a single heavy chain variable region, is one-tenth of the size of traditional monoclonal antibodies. It possesses outstanding tissue permeability (Sun et al., 2021) and is able to overcome the limitations related to antibody injection, thus achieving a non-invasive administration. Remarkably, nanobody exhibits lower immunogenicity (Sun et al., 2021), which renders it more applicable to the unique immune environment of the eye. Bispecific nanobody is capable of simultaneously binding two specific epitopes or proteins, thereby performing special functions and attaining biological functions that monoclonal antibody drugs cannot achieve. It demonstrates greater specificity, targeting capacity, and lower off-target toxicity, and exhibits advantages in blocking target signals, reducing immune escape, and combating drug resistance (Wang et al., 2021).

In the present study, the objective is to design a bispecific nanobody, Nb-TV, which concurrently targets TNF-α and VEGF-A to fulfill the “anti-inflammation and anti-angiogenesis” dual-target strategy. It is intended to assess the anti-inflammatory and anti-angiogenic activities of Nb-TV *in vitro* through its action on the inflammatory model of ARPE-19 and the angiogenic model of HUVEC. Additionally, the *in vivo* therapeutic potential of Nb-TV for DR is expected to be evaluated by constructing a streptozotocin-induced diabetic mouse retinopathy model. This study will furnish a significant theoretical foundation for the utilization of TNF-α and VEGF-A as therapeutic targets in DR and for the treatment of retinopathy via non-invasive administration of nanobodies.

## 2 Materials and methods

The handling of mice and experimental procedures were conducted in accordance with national and institutional guidelines for animal care and were approved the Research Ethics Committees of East China University of Science and Technology (Shanghai, China), as approved by the mentioned committees (REC No. 20181223).

### 2.1 Generation of bispecific nanobody Nb-TV

The gene sequences of anti-TNF-α nanobody (Nb-TNFα) and anti-VEGF-A nanobody (Nb-VEGFA) were synthesized by Tsingke Biotechnology (Shanghai, China). The sequence information of Nb-TNFα and Nb-VEGFA were provided by our laboratory. The bispecific nanobody Nb-TV was obtained by linking Nb-TNFα and Nb-VEGFA via a G4S flexible linker. The TNF-α, VEGF-A, Nb-TNFα, Nb-VEGFA, and Nb-TV sequence were inserted into the pET-24a plasmid through the *Nde* I and *Xho* I restriction sites and then confirmed by sequencing, respectively. The constructed plasmid was then transferred into *E. coli* Rosetta gami2 (DE3) (Cat #EC1013, Weidibio, Shanghai, China). A 3% inoculum was inoculated into 1 L of culture. When the OD600 value of the culture reached 0.6, induction was carried out with 0.4 mM IPTG (Cat #367–93–1, Macklin, Shanghai, China) at 37 °C for 10 h. Subsequently, purification was performed using Ni-NTA affinity chromatography. The purified proteins were verified through sodium dodecyl sulfate-polyacrylamide gel electrophoresis (SDS-PAGE).

### 2.2 Protein structure simulation and molecular docking

The sequence of Nb-TV was uploaded to I-TASSER for simulating its three-dimensional structure. Meanwhile, the sequences of Nb-TV, TNF-α, and VEGF-A were separately uploaded to the HDOCK server to simulate the docking scenarios of Nb-TV with TNF-α and of Nb-TV with VEGF-A.

### 2.3 Enzyme-linked immunosorbent assay

Immune plate was coated with antigen (diluted with Na_2_CO_3_ (Cat #S818014, Macklin, Shanghai, China) buffer, pH9.6) at a concentration of 10 μg/mL per well overnight at 4 °C. After blocking with 5% skim milk (Cat #S917757, Macklin, Shanghai, China) at 37 °C for 1 h, Nb-TV, Nb-TNFα, and Nb-VEGFA diluted in phosphate buffered saline buffer (PBS) (Cat #P750064, Macklin, Shanghai, China) were added to the plate in a dilution series and then incubated at 37 °C for 2 h. After washing, HRP Anti-HA tag antibody (Cat #100028-MM10-H, Sinobiological, Beijing, China) was incubated at 37 °C for 2 h as a secondary antibody. After incubation with TMB (Cat #PR1200, Solarbio, Beijing, China), the reaction was terminated by H_2_SO_4_ (Cat #81936C, Adamas-life, Shanghai, China). The absorbance (OD) of each well was detected at 450 nm.

### 2.4 Cell culture

ARPE-19 cells were cultivated in DMEM/F12 medium (Cat #D6501, Solarbio, Beijing, China) supplemented with 10% FBS and 1% streptomycin-penicillin-amphotericin B. HUVEC cells were cultured in DMEM medium with 10% fetal bovine serum (FBS) (Cat #FS201-02, Transgen, Beijing, China) and 1% streptomycin-penicillin-amphotericin B (Cat #P7630, Solarbio, Beijing, China). The culture was maintained at 37 °C under a 5% CO_2_ atmosphere. The medium was refreshed every 2 days. The cells were detached and passaged using 0.25% trypsin-EDTA (Cat #11995, Solarbio, Beijing, China) in accordance with their actual growth status.

### 2.5 Cell viability MTT assay

ARPE-19 (1.5 × 10^4^) cells were seeded into 96-well plate and incubated overnight. TNF-α (0.53 μM) was added to model and drug groups. The drug group received a dilution series of Nb-VEGFA, Nb-TNFα or Nb-TV. After 24 h incubation, 3-(4,5-Dimethylthiazol-2-yl)-2,5-diphenyltetrazolium bromide (MTT) (Cat #T6126, Macklin, Shanghai, China) was added to each well for 4 h. Then, a 150 μL of DMSO (Cat #D806645, Macklin, Shanghai, China) was added at room temperature for 15 min. OD490 nm was measured using a microplate reader. HUVEC (2 × 10^4^) cells were seeded in 96-well plate and incubated overnight. VEGF-A (0.02 μM) was added to model and drug groups. The drug group received a dilution series of Nb-VEGFA, Nb-TNFα or Nb-TV. Subsequent steps are as described above.

### 2.6 Cell immunofluorescence staining

ARPE-19 (8 × 10^3^) cells were seeded in 96-well plate, and co-treated with Nb-TNFα or Nb-VEGFA or Nb-TV and TNF-α for 10 h. Cells were fixed and permeabilized with 4 °C pre-cooled anhydrous methanol for 15 min, then blocked with 1% bovine serum albumin (BSA) (Cat #SW3015, Solarbio, Beijing, China) at 37 °C for 1 h, and incubated with Rabbit anti-NF-κB p65 antibody (Cat #ER0815, HUABIO, Hangzhou, China) diluted in 1% BSA at 4 °C overnight. Goat Anti-Rabbit IgG H&L (Alexa Fluor® 488) (Cat #ab150077, Abcam, United Kingdom) as secondary antibody. The nuclear was stained with DAPI (Cat #C0060, Solarbio, Beijing, China) for 10 min at room temperature. Images were captured using a fluorescence inverted microscope (XDS-200C, Olympus, Japan). For each group, at least four random fields of view (>100 cells) were collected, and the average fluorescence intensity of the images was analyzed using ImageJ software. HUVEC (5 × 10^3^) cells were seeded in 96-well plate, and co-treated with Nb-TNFα or Nb-VEGFA or Nb-TV and TNF-α for 10 h. Primary antibodies were Rabbit anti-p38 alpha/MAPK14 k antibody (Cat #ET1602-26 HUABIO, Hangzhou, China) and iNOS polyclonal antibody (Cat #YP-Ab17172, UpingBio, Hangzhou, China). The following steps are the same as above.

### 2.7 Quantitative real-time polymerase chain reaction

ARPE-19 were seeded in 6-well plate. Cells were co-treated with Nb-TNFα or Nb-VEGFA or Nb-TV and TNF-α for 24 h. Total RNA extracted from the cells was reverse-transcribed into cDNA using BeyoRT™ III cDNA First Strand Synthesis System (Cat #D7178S, Beyotime, Shanghai, China). qPCR analysis was performed with 2×Color SYBR Green qPCR Master Mix (Low ROX) (Cat #G8047-1, Adamas-life, Shanghai, China). The primers used are shown as follows: for *IL-6*, forward 5′-TCT​GCG​CAG​CTT​TAA​GGA​GT-3′ and reverse 5′-CCC​AGT​GGA​CAG​GTT​TCT​GA-3′; for *IL-8*, forward 5′-TACTCCAAAC CTTTCCACCCC-3′ and reverse 5′-CCC​AGT​TTT​CCT​TGG​GGT​CC-3′; for *MCP-1*, forward 5′-CAA​GCA​GAA​GTG​GGT​TCA​GG-3′ and reverse 5′-TGG​GGA​AAG​CTA​GGG​GAA​AAT-3′; for *GAPDH*, forward 5′-CCA​CTC​CTC​CAC​CTT​TGA​CG-3′ and reverse 5′-TAG​CCA​AAT​TCG​TTG​TCA​TAC​CAG​G -3′.

### 2.8 Western blot

ARPE-19 were treated with VEGF-A and different nanobodies: Nb-TNFα, Nb-VEGFA, and Nb-TV respectively for 24 h. The cells were harvested and lysed with radioimmunoprecipitation assay buffer (RIPA) (1% phenylmethanesulfonyl fluoride (PMSF)) (Cat #R0010, Solarbio, Beijing, China) on ice for 30 min. The protein-containing supernatant was collected by centrifugation at 12400 *g* at 4 °C. 30 μg of each sample was subjected to SDS-PAGE and transferred onto a PVDF membrane (Cat #FFP24, Beyotime, Shanghai, China). The PVDF membrane was blocked with 5% skim milk powder in TBST buffer (Cat #T917679, Macklin, Shanghai, China) at room temperature for 2 h and then incubated with the following primary antibodies: anti GAPDH (Cat #10494-1-AP, Proteintech, Chicago, United States), anti-IL-6 (Cat #WL02841, Wanleibio, Shenyang, China), anti-IL-8 (Cat #WL03074, Wanleibio, Shenyang, China), anti MCP-1 (Cat #P13500, CUSABIO, Wuhan, China) and anti-Caspase-1 (Cat #ET1608-69, HUABIO, Hangzhou, China) overnight at 4 °C, followed by incubation with the diluted secondary antibody at room temperature for 1 h. Color exposure was performed using the ECL kit (Cat #36208 ES, Yeasen, Shanghai, China) and photographed with a gel imaging system. ImageJ was used to analyze the grayscale of the bands.

HUVEC were treated with VEGF-A and different nanobodies: Nb-TNFα, Nb-VEGFA, and Nb-TV respectively for 24 h. Primary antibodies were Anti-ERK1/2 rabbit pAb (Cat #WL01864, Wanleibio, Shenyang, China), Anti-ERK1 (phospho-T202/Y204) + ERK2 (phospho-T185/Y187) antibody (Cat #BM3950, BOSTER, Wuhan, China) and GAPDH (Cat #10494-1-AP, Proteintech, Chicago, United States). The following steps are the same as before.

### 2.9 Scratch assay

HUVEC were seeded in 12-well plates. Once the cells grew to contact inhibition, a scratch was created using a 200 μL pipette tip. The floating cells were removed by washing with PBS. Cells were co-treated with Nb-TNFα or Nb-VEGFA or Nb-TV and VEGF in basal medium (containing 1% FBS), and the cells were further cultured for 36 h. Photographs were taken at 0 h, 12 h, 24 h, and 36 h.

### 2.10 Angiogenesis assay

A 96-well cell culture plate was coated with 50 μL of growth factor-reduced Mogengel Matrix (Cat #AC-M082703, ACROBiosystems, Beijing, China) and solidified at 37 °C for 1 h. HUVEC cells were seeded on the plate at a density of 3 × 10^4^. The experiment was carried out in the presence of VEGF-A and different nanobodies: Nb-TNFα, Nb-VEGFA and Nb-TV, respectively. The cells were further incubated for 4 h. The angiogenesis situation was analyzed by the ImageJ plugin Angiogenesis Analyzer.

### 2.11 Animal grouping and drug administration

Thirty 6-week-old male C57BL/6 mice (Cat #N000013, Gempharmatech, Shanghai, China) were randomly divided into a control group (*n* = 6) and a diabetic retinopathy (DR) group (*n* = 24). After a 6-h fasting period, the mice in the DR group were intraperitoneally injected with streptozotocin (STZ) (Cat #S6089, Macklin, Shanghai, China) freshly dissolved in sodium citrate (Cat #S818273, Macklin, Shanghai, China) buffer (pH 4.2–4.5) at a dose of 55 mg/kg for 5 consecutive days. The control group received an equal volume of sodium citrate solution via intraperitoneal injection. After the final injection, blood glucose levels were regularly monitored. The successful establishment of the DR model was indicated when the blood glucose value exceeded 16.7 mmol/L. Mice in DR group and control group were fed normal diet for 2 months. Then the DR group was randomly divided into DR group (eye drop PBS), Nb-TNF-α group (eye drop 0.2 mg/mL Nb-TNFα), Nb-VEGFA group (eye drop 0.2 mg/mL Nb-VEGFA) and Nb-TV group (eye drop 0.2 mg/mL Nb-TV). The control group received the same amount of PBS. Retinas were harvested and therapeutic efficacy was evaluated at 2 weeks and 4 weeks after nanobody treatment, respectively.

### 2.12 Periodic acid-schiff (PAS) staining

Under a dissecting microscope, the retina was carefully dissected. An appropriate amount of digestive enzyme solution was added, and enzymatic digestion was carried out at 37 °C for 30 min. Subsequently, centrifugation was performed to remove the enzyme and excess tissues. The retina was evenly spread on glass slides and dried at a low temperature. The slides were immersed in an iodic acid solution for oxidation for 10 min, then placed in Schiff’s reagent for staining in the dark for 30 min. Next, the slides were put into hematoxylin staining solution for staining for 10 min. Subsequently, the slides were immersed in a differentiation solution for 20 s, and then placed in a bluing solution for 2 min until the cell nuclei turned blue. The slides were placed successively in 70%, 80%, 90%, 95%, and 100% alcohol for dehydration. An appropriate amount of mounting medium was dropped on the slides and they were covered with coverslips. Finally, they were observed under a microscope.

### 2.13 Mice retinal paraffin sections

The eyeballs of mice were harvested and fixed in an eyeball fixative solution (Cat #G1109, Servicebio, Wuhan, China) for 24 h. Subsequently, the fixed eyeballs were dehydrated and embedded in paraffin. Sections with a thickness of 4 μm were cut parallel to the eye axis, flattened in 45 °C warm water, picked up with anti-drop slides, baked (60 °C) in an oven for 6 h, and then prepared for the next procedure.

### 2.14 Immunofluorescence assay

The paraffin sections of the retina were blocked with a 3% bovine serum albumin (BSA) solution at room temperature for 30 min. The Anti-TNF-alpha Rabbit pAb (Cat #GB11188, Servicebio, Wuhan, China) and recombinant Anti-VEGFA antibody (Rabbit mAb) (Cat #GB15165, Servicebio, Wuhan, China), which were diluted in PBS, were added and incubated overnight at 4 °C. After being washed three times with PBS, the diluted fluorescent-labeled secondary antibodies were added and incubated at room temperature in the dark for 50 min. The cell nuclei were stained with DAPI solution and incubated at room temperature in the dark for 10 min. Finally, the fluorescent images were captured using a scanner.

### 2.15 Hematoxylin and eosin (H&E) staining

Paraffin sections were first subjected to deparaffinization in a dilution series of xylene (Cat #X821391, Macklin, Shanghai, China) solutions. Subsequently, the sections were rehydrated step-by-step in a dilution series of ethanol (Cat #E821482, Macklin, Shanghai, China) solutions and finally through distilled water. Hematoxylin staining was then performed on the sections, followed by a 20-min rinse with tap water. After that, eosin staining was carried out. The stained sections were dehydrated using a series of alcohol solutions with increasing concentrations. Post-dehydration, the sections were dried in a fume hood until they became transparent. Then, neutral gum (Cat #N861409, Macklin, Shanghai, China) was applied to the center of the tissue for mounting, and the mounted sections were further dried in the fume hood. Finally, the retinal sections were examined under a light microscope.

### 2.16 Statistical analysis

Data were statistically analyzed and presented using Prism 9.5 (GraphPad Software, San Diego, CA, United States). Data were presented as mean ± standard deviation (SD) from at least three independent experiments, each performed on different days with distinct cell passages. Each independent experiment included at least three technical replicates per condition. The two-sample t-test was used for comparisons between two groups. One-way ANOVA was used for comparisons among multiple groups, followed by the Tukey’s *post hoc* test. *: compared with negative control; #: compared with TNF-α/VEGF-A treated control. *P* value of <0.05 was considered significant.

## 3 Result

### 3.1 Generation of bispecific nanobody Nb-TV

**FIGURE 1:**
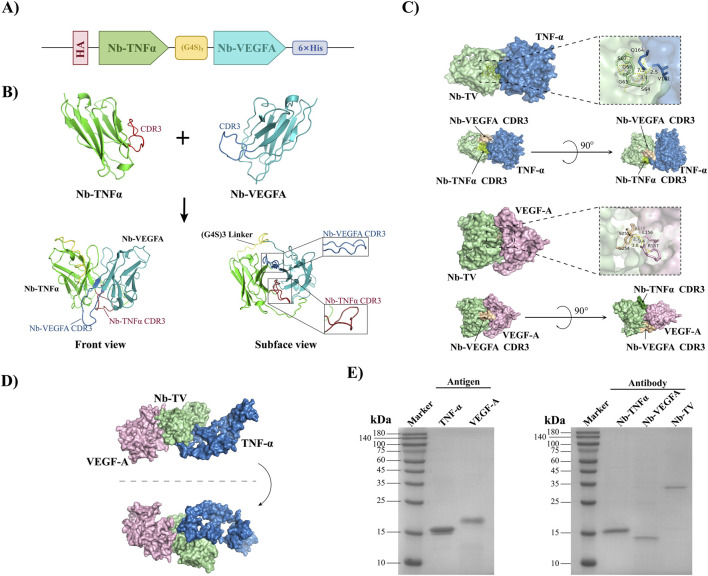
Design, simulation and generation of bispecific nanobody Nb-TV. **(A)** Schematic diagram of recombinant plasmid of Nb-TV. **(B)** Structure diagram of Nb-TV. Green: Nb-TNFα. Blue: Nb-VEGFA. **(C)** Schematic diagram of Nb-TV in combination with VEGF or TNF-α. The online tool HDOCK was used for docking. **(D)** Schematic diagram of Nb-TV in combination with VEGF and TNF-α simultaneously. **(E)** Purification of TNF-α, VEGF-A, Nb-TNFα, Nb-VEGFA and Nb-TV. TNF-α: 18.5 kDa. VEGF-A: 20.4 kDa. Nb-TNFα: 15.9 kDa. Nb-VEGFA: 14.9 kDa. Nb-TV: 30.6 kDa.

As presented in Figure 1A, the schematic diagram of Nb-TV construction was illustrated. Nb-TNFα was located at the N-terminus, while Nb-VEGFA was situated at the C-terminus, with three repeated G4S serving as the linker in between, and the His-Tag at the C-terminal end being utilized as the purification tag. Figure 1B respectively displayed the three-dimensional structures of Nb-TNFα, Nb-VEGFA and Nb-TV simulated by I-TASSER (Figure 1B). It was observed that the structures of Nb-TNFα and Nb-VEGFA within Nb-TV exhibited no significant differences from those of the individual Nb-TNFα and Nb-VEGFA. Subsequently, the HDOCK server was employed to simulate the docking of Nb-TV with TNF-α or VEGF-A (Figure 1C). It was found that Nb-TV could bind to either TNF-α or VEGF-A alone. When both antigens were present simultaneously, Nb-TV was still able to bind to VEGF-A even after binding to TNF-α, suggesting that Nb-TV possessed dual-targeting activity (Figure 1D). The SDS-PAGE results indicated that the purified TNF-α, VEGF-A, Nb-TNFα, Nb-VEGFA and Nb-TV presented single bands, and their purities were all greater than 95% (Figure 1E).

### 3.2 The binding activity of Nb-TV to TNF-α and VEGF-A

**FIGURE 2:**
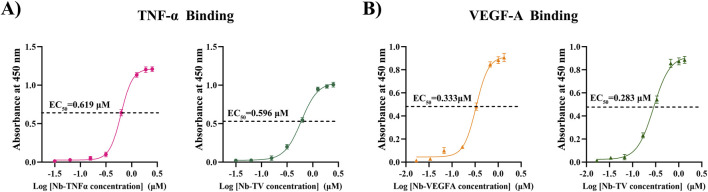
The binding activity of Nb-TV to TNF-α and VEGF-A was detected by ELISA. **(A)** Binding activity of Nb-TV and Nb-TNFα to TNF-α. EC_50_ (Nb-TNFα, TNF-α) = 0.619 μM, EC_50_ (Nb-TV, TNF-α) = 0.596 μM. **(B)** Binding activity of Nb-TV and Nb-VEGFA to VEGF-A. EC_50_ (Nb-VEGFA, VEGF-A) = 0.333 μM, EC_50_ (Nb-TV, VEGF-A) = 0.283 mM.

The binding capacity of Nb-TV was assessed via indirect ELISA. In the case of the antigen TNF-α (as shown in Figure 2A), the *Kd* value of Nb-TNFα was determined to be 0.619 μM, while that of Nb-TV was 0.596 μM. For the antigen VEGF-A (depicted in Figure 2B), the affinity *Kd* value of Nb-VEGFA was 0.333 μM and that of Nb-TV was 0.283 μM. The binding capacity of Nb-TV to TNF-α was 1.04 times greater than that of Nb-TNFα, and its binding capacity to VEGF-A was 1.18 times higher than that of Nb-VEGFA. These results suggest that the bispecific nanobody not only enhances specificity but also guarantees that the binding activity of the internal single-targeted nanobodies to their respective target antigens remains unaltered.

### 3.3 Nb-TV improves TNF-α-induced inflammation in ARPE-19 cells

**FIGURE 3:**
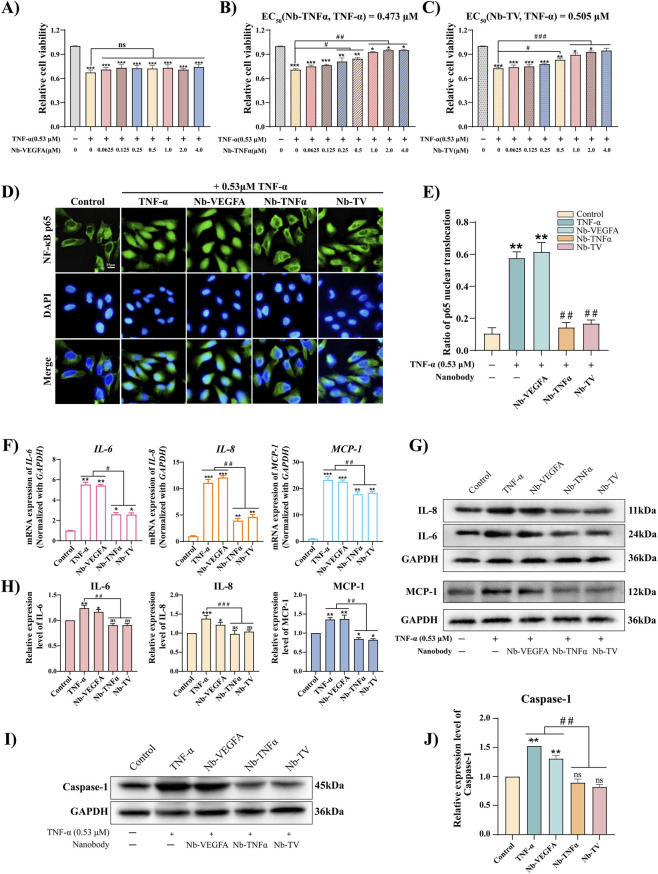
Nb-TV inhibits TNF-α-induced inflammation in ARPE-19 cells. **(A–C)** MTT assay detected the antagonism of Nb-VEGFA, Nb-TNFα and Nb-TV to TNF-α in ARPE-19 (*n* = 5). **(D,E)** Immunofluorescence detected the inhibitory effect of Nb-TV, Nb-TNFα and Nb-VEGFA on the nuclear translocation of p65 in ARPE-19 induced by TNF-α. Green: p65; blue: DAPI (*n* = 3, Scale = 50 μm). **(F)** qPCR detected the relative change of IL-6, IL-8 and MCP-1 mRNA expression in TNF-α-induced ARPE-19 cells treated with Nb-TV and Nb-VEGFA (*n* = 5). **(G,H)** Western blot detected the protein expression level of IL-6, IL-8 and MCP-1 in TNF-α-induced ARPE-19 treated with Nb-TV, Nb-TNFα and Nb-VEGFA (*n* = 3). **(I,J)** Western blot detected the protein expression level of Caspase-1 in TNF-α-induced ARPE-19 treated with Nb-TV, Nb-TNFα and Nb-VEGFA (*n* = 3). Values are presented as mean ± SD; ^*^ *P* < 0.05, ^**^ *P* < 0.01, ^***^ *P* < 0.001 compared with Control group; ^#^ *P* < 0.05, ^##^ *P* < 0.01, ^###^ *P* < 0.001 compared with TNF-α group.

TNF-α, an early inflammatory mediator, activates inflammation-related pathways, triggers reactions, causes cell damage and cycle arrest, inhibiting proliferation (Subhi and Lykke Sørensen, 2017). Upon treatment with 0.53 μM TNF-α on ARPE-19, a significant reduction in cell activity was observed, with a decrease of approximately 30%. Nb-VEGFA, which lacks binding affinity for TNF-α, failed to ameliorate the TNF-α-induced cell proliferation inhibition (Figure 3A). In contrast, under the influence of Nb-TV and Nb-TNFα, the cell viability exhibited a concentration-dependent increase (Figures 3B,C). At a concentration of 0.025 μM Nb-TNFα (Figure 3B), a remarkable improvement in ARPE-19 cell viability was noted, with a half-maximal effective concentration of 0.473 μM. Under identical experimental conditions, 0.5 μM Nb-TV effectively restored the TNF-α-induced inhibition of ARPE-19 cell proliferation. The half-maximal effective concentration was determined to be 0.505 μM, and the final cell activity was comparable to that of the control group (Figure 3C). The calculated effect ratio between Nb-TV and Nb-TNFα was 1.07, indicating equivalent efficacy in reversing the TNF-α-induced ARPE-19 cell proliferation inhibition, and both treatments demonstrated significant effects.

TNF-α functions by activating the NF-κB signaling pathway. The activated NF-κB is released from the NF-κB/IκB complex and translocate into the nucleus, thereby regulating the transcription of downstream genes. The inhibitory effect of Nb-TV on the TNF-α/NF-κB signaling pathway was analyzed by immunofluorescence assay (Figures 3D,E). In the control group, p65 was mainly distributed in the cytoplasm of ARPE-19 cells. Under TNF-α stimulation alone, p65 underwent nuclear translocation, and the proportion of nuclear p65 to the total cellular p65 was 57.73%, indicating the activation of the NF-κB pathway. When co-stimulated with Nb-TNFα or Nb-TV and TNF-α, the nuclear translocation of p65 was significantly inhibited, and the proportions of nuclear p65 were only 14.34% and 16.83%, respectively. These results suggest that both Nb-TNFα and Nb-TV are capable of blocking the activation of the NF-κB signaling pathway by TNF-α. TNF-α activates the NF-κB pathway, thereby leading to the upregulation of the expression of downstream genes IL-6, IL-8 and MCP-1. These cytokines are involved in both inflammatory responses and angiogenesis. As determined by qPCR and Western blot, treatment with TNF-α caused a significant increase in the levels of IL-6, IL-8 and MCP-1 in ARPE-19 cells. In contrast, in the groups co-treated with Nb-TNFα or Nb-TV and TNF-α, the levels of IL-6, IL-8 and MCP-1 were markedly reduced at both transcriptional and translational levels. These results indicate that Nb-TV is capable of ameliorating the inflammatory response (Figures 3F–H).

In an inflammatory environment, activation of the NF-κB/NLRP3/Caspas-1 pathway induces retinal pigment epithelial cell apoptosis and impairs retinal barrier integrity (Lu et al., 2018; Hao et al., 2019). Western blot analysis of Caspase-1 expression in ARPE-19 cells revealed that TNF-α treatment significantly upregulated its expression. In contrast, when co-treated with Nb-TNFα or Nb-TV, the Caspase-1 level was remarkably lower than that in the TNF-α alone group (Figures 3I,J). These results indicate that Nb-TV is able to effectively protect the survival of ARPE-19 cells in an inflammatory environment. Overall, Nb-TV exerts an anti-inflammatory effect by targeting and binding to TNF-α, which inhibits the activation of the NF-κB inflammatory pathway and reduces the release of IL-6, IL-8, and MCP-1. Meanwhile, it suppresses the destruction of the retinal pigment epithelium by blocking the NF-κB/NLRP3/Caspas-1 pathway.

### 3.4 Nb-TV inhibits VEGF-a-induced HUVEC hyperproliferation and migration

**FIGURE 4:**
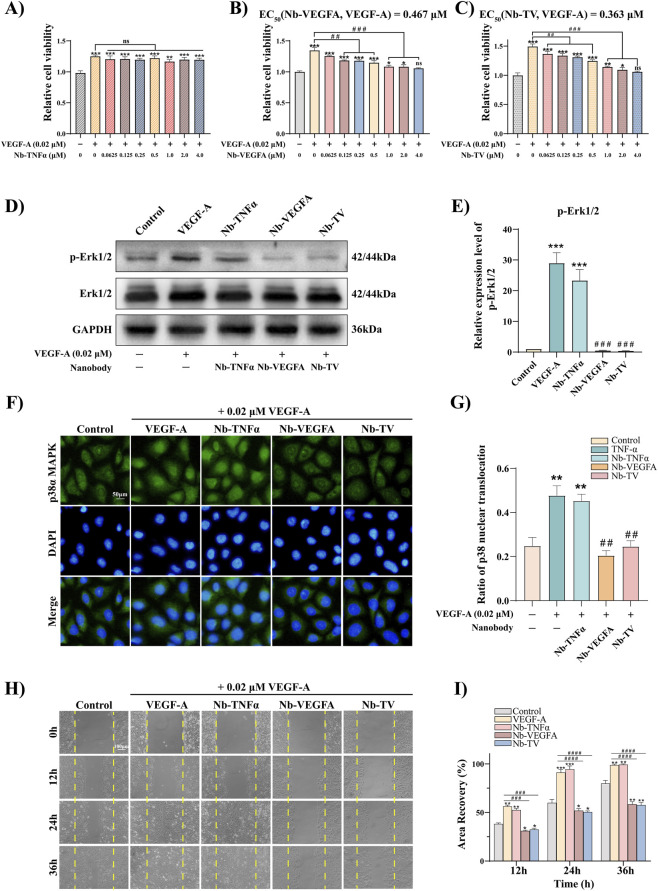
Nb-TV inhibits VEGF-A-induced HUVEC hyperproliferation and hypermigration. **(A–C)** MTT assay detected the antagonism of Nb-TNFα, Nb- VEGFA and Nb-TV to VEGF-A in HUVEC (*n* = 5). **(D,E)** Western blot detected the protein expression level of p-Erk1/2 and Erk1/2 in VEGF-A -induced HUVEC treated with Nb-TV, Nb-TNFα and Nb-VEGFA (*n* = 3). **(F,G)** Immunofluorescence detected the inhibitory effect of Nb-TV, Nb-TNFα and Nb-VEGFA on the nuclear translocation of p38α in HUVEC induced by VEGF-A. Green: p38α; blue: DAPI (*n* = 3, Scale = 50 μm). **(H,I)** Scratch test detected the migration ability of VEGF-A -induced HUVEC treated with Nb-TV, Nb-TNFα and Nb-VEGFA (*n* = 3, Scale = 100 μm). Values are presented as mean ± SD; ^*^ *P* < 0.05, ^**^ *P* < 0.01, ^***^ *P* < 0.001 compared with Control group; ^##^ *P* < 0.01, ^###^ *P* < 0.001, ^####^ *P* < 0.0001 compared with VEGF-A group.

VEGF-A is essential in angiogenesis, binding to VEGFR2 on vascular endothelium to initiate related pathways. The activation of these signal pathways promotes the cell cycle and cell proliferation. The MTT results showed that treatment with VEGF-A for 24 h remarkably promoted the proliferation of HUVEC cells. Different concentrations of Nb-TNFα had no influence on the proliferation-promoting effect of VEGF-A (Figure 4A). In contrast, both Nb-VEGFA and Nb-TV had inhibitory effects on the proliferation-promoting effect of VEGF-A in a concentration-dependent manner. The half-maximal effective concentration of Nb-VEGFA was 0.467 μM, and that of Nb-TV was 0.363 μM (Figures 4B,C).

VEGF-A promotes cell proliferation and participates in angiogenesis through Erk1/2 signal transduction. Western blot was utilized to analyze the phosphorylation levels of Erk1/2 in HUVEC cells under different treatment conditions. The level of phosphorylated Erk1/2 in the VEGF-A group was 30 times that of the control group. In contrast, the phosphorylation levels of Erk1/2 in the groups co-treated with VEGF-A and Nb-VEGFA or Nb-TV were lower than that of the control group, which indicates that Nb-TV can effectively inhibit VEGF-A-induced vascular cell proliferation (Figures 4D,E).

Endothelial cell migration is a crucial step in new blood vessel formation. The binding of VEGF-A to vascular endothelial growth factor receptor 2 (VEGFR2) triggers the activation of the p38 MAPK pathway, resulting in the nuclear translocation of phosphorylated p38, which subsequently promotes endothelial cell migration and maintains the structure and function of blood vessels. Immunofluorescence assays were employed to analyze the cellular distribution of phosphorylated p38 in HUVECs after different treatments (Figures 4F,G). In the control group, phosphorylated p38 was mainly distributed in the cytoplasm. After 24-h exposure to VEGF-A, the nuclear level of phosphorylated p38 was remarkably elevated and was double that of the control. Nb-TNFα exerted no influence on the nuclear translocation of phosphorylated p38 induced by VEGF-A. The binding of Nb-VEGFA or Nb-TV with VEGF-A impeded the binding of VEGF-A to VEGFR2, thus suppressing the nuclear translocation of phosphorylated p38, and the nuclear level of phosphorylated p38 was comparable to that of the control. This finding was further corroborated by scratch assays (Figures 4H,I). VEGF-A markedly enhanced HUVEC cell migration, whereas Nb-VEGFA and Nb-TV counteracted the migration-stimulating activity of VEGF-A.

### 3.5 Nb-TV inhibited the angiogenesis of HUVEC cells *in vitro* and decreased vascular permeability

**FIGURE 5:**
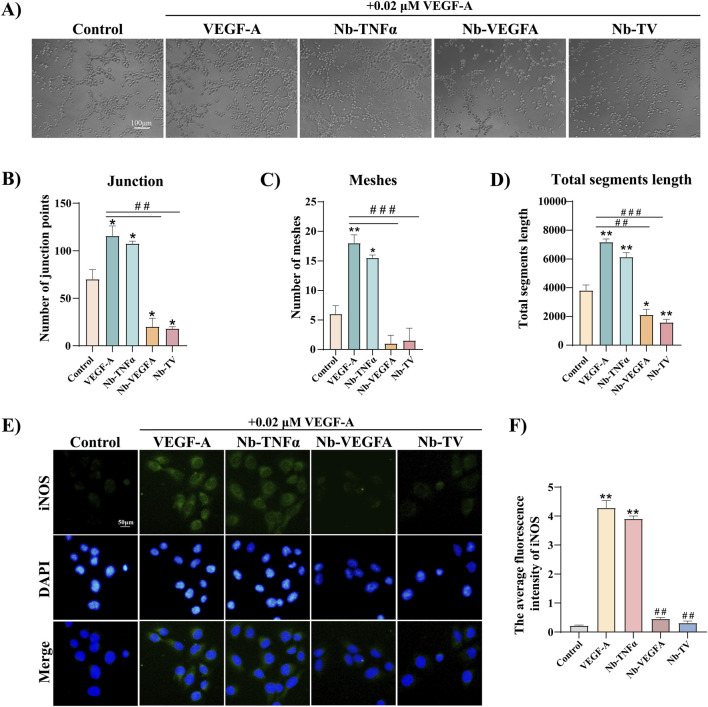
Nb-TV inhibits VEGF-A-induced angiogenesis of HUVEC *in vitro* and reduces vascular permeability. **(A–D)** Tube formation assay detected the angiogenesis ability of VEGF-A-induced HUVEC treated with Nb-TV, Nb-TNFα and Nb-VEGFA (*n* = 3, Scale = 100 μm). **(E,F)** Immunofluorescence detected the inhibitory effect of Nb-TV, Nb-TNFα and Nb-VEGFA on the iNOS expression in HUVEC induced by VEGF-A. Green: iNOS; blue: DAPI (*n* = 3, Scale = 50 μm). Values are presented as mean ± SD; ^*^ *P* < 0.05, ^**^ *P* < 0.01, compared with Control group; ^##^ *P* < 0.01, ^###^ *P* < 0.001, compared with VEGF-A group.

To clarify the anti-angiogenic effect of Nb-TV, a tube formation assay was conducted (Figure 5A). Upon exposure to VEGF-A, the number of junction points of HUVECs was augmented by 64.3% (Figure 5B), the number of meshes was doubled (Figure 5C), and the total segment length was increased by 88.3% (Figures 5C,D), suggesting that VEGF-A could remarkably promote the *in vitro* angiogenesis of HUVECs. With the treatment of Nb-VEGFA or Nb-TV, the migration of HUVECs was suppressed and angiogenesis was considerably diminished, with merely 1-2 meshes being formed (Figure 5C), and the total segment length was also notably shortened (Figure 5D). Nb-TV reduced the number of junction points by 84.4%, the number of meshes by 91.7%, and the total segment length by 80%, powerfully evidencing that Nb-TV possesses the inhibitory capacity against the *in vitro* angiogenesis of HUVECs induced by VEGF-A.

During the progression of PDR, in addition to the emergence of neovascularization, a significant increase in vascular permeability can be observed. The binding of VEGF-A to VEGFR2 modulates vascular permeability through nitric oxide synthase (NOS). High levels of VEGF-A result in an increase in the expression of iNOS, which leads to an elevation in vascular permeability and subsequently undermines vascular integrity. As illustrated in Figures 5E,F, the expression of iNOS in HUVECs treated with VEGF-A was upregulated by 20 times. This will cause an increase in vascular permeability and is unfavorable for the maintenance of the normal structure of blood vessels. Meanwhile, in the groups treated with Nb-VEGFA and Nb-TV, the expression level of iNOS was basically the same as that in the control group, suggesting that after binding to VEGF-A, Nb-VEGFA and Nb-TV can effectively maintain the permeability of blood vessels.

### 3.6 Nb-TV can effectively improve diabetic retinopathy *in vivo*

**FIGURE 6:**
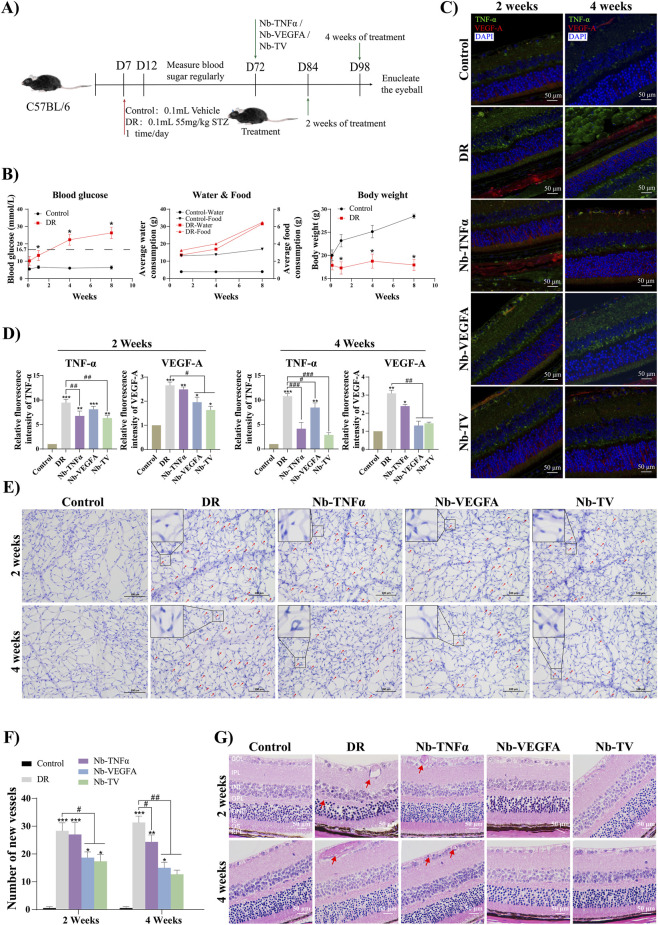
Effects of Nb-TV improved retinopathy in DR mice. **(A)** Schematic diagram of the establishment of the STZ - induced diabetic retinopathy model in mice and the intervention of Nb-TV, Nb-TNFα and Nb-VEGFA. **(B)** Fasting blood glucose, diet, and body weight of mice in the control group and DR group after the injection of STZ. **(C,D)** Immunofluorescence detected the inhibitory effect of Nb-TV, Nb-TNFα and Nb-VEGFA on TNF-α and VEGF-A in the retinas of DR mice. Green: TNF-α; red: VEGF-A; blue: DAPI (*n* = 6, Scale = 50 μm). **(E,F)** PAS staining of retinal vascular network flat-mounts detected the improving effect of Nb-TV, Nb-TNFα and Nb-VEGFA on retinal angiogenesis in DR mice (*n* = 6, Scale = 100 μm). **(G)** HE staining detected the improving effect of Nb-TV, Nb-TNFα and Nb-VEGFA on retinal pathological changes in DR mice (*n* = 6, Scale = 50 μm). Values are presented as mean ± SD; ^*^ *P* < 0.05, ^**^ *P* < 0.01, ^***^ *P* < 0.001 compared with Control group; ^#^ *P* < 0.05, ^##^ *P* < 0.01, ^###^ *P* < 0.001, compared with DR group.

Animal experiments were conducted in accordance with the design presented in Figure 6A. After continuous injection of STZ, the mice in the DR group manifested typical diabetic characteristics (Figure 6B). Compared with the control group, their blood glucose levels increased steadily and exceeded 16.7 mmol/L in the second week. Meanwhile, their food intake increased significantly, yet their body weight remained almost unchanged.

After 2-week and 4-week treatments, the Nb-TV group demonstrated the optimal therapeutic effect, with significantly decreased levels of TNF-α and VEGF-A in the retinas of the mice (Figures 6C,D). Compared to the DR group, after 2 weeks of treatment, the TNF-α level decreased by 30.47%, and the VEGF-A level decreased by 35.48%. After 4 weeks of treatment, the TNF-α level decreased by 73.29%, and the VEGF-A level decreased by 53.63%. Simultaneously, treatment with Nb-TNFα led to a 61.26% reduction in TNF-α, and treatment with Nb-VEGFA resulted in a 57.44% decrease in VEGF-A.

Notably, during the two - week treatment period, Nb-TNFα had no impact on the VEGF-A level, and Nb-VEGFA had no effect on the TNF-α level. However, after 4 weeks of treatment, Nb-TNFα caused a 22.86% decrease in the VEGF-A level, and Nb-VEGFA led to a 19.44% reduction in the TNF-α level. This indicates a close relationship between TNF-α and VEGF-A, suggesting that the inhibition of one factor can influence the other.

Furthermore, angiogenesis was observed by means of PAS staining. As shown in Figure 6E, compared with the control group, a large number of non - functional grown angiogenesis (red arrows) were generated in the retinas of the DR group. After 2 weeks of treatment with Nb-TV, the number of new blood vessels decreased by 38.83% compared with the DR group (Figures 6E,F). At the same time, Nb-VEGFA decreased the number of new blood vessels by 34.10%, while Nb-TNFα did not show the effect of inhibiting angiogenesis. After 4 weeks of treatment, Nb-TV effectively reduced the number of new blood vessels by 58.06%, Nb-VEGFA reduced it by 51.60%, and Nb-TNFα reduced it by 19.35%.

Finally, the pathological structure of the retinal tissues in mice was analyzed by HE staining (Figure 6G). In the retinas of the control group mice, the tissue structure was clear, the ganglion cells were arranged closely, and the inner nuclear layer (INL) and outer nuclear layer (ONL) were intact and compact. In the retinas of the DR group, the inner and outer nuclear layers were loose, the cells were arranged disorderly, and dilated new blood vessels (red arrows) could be seen. After 2 weeks of treatment with Nb-TV, the above-mentioned pathological changes were improved. After 4 weeks of treatment, the diseased retinas were effectively restored.

In conclusion, Nb-TV effectively reduces the levels of TNF-α and VEGF-A in the retinas of DR mice, decreases retinal angiogenesis, and improves the pathological changes of the retinal structure. Based on the above results, it is considered that Nb-TV has an effective therapeutic effect on DR.

## 4 Discussion

Diabetic retinopathy is one of the prevalent microvascular complications of diabetes mellitus. It is manifested by retinal microhemangioma, increased permeability of retinal capillary, retinal hemorrhage and exudation, and neovascularization. In the condition of long-term hyperglycemia, retinal oxidative stress is enhanced, angiogenic factors and inflammatory response are unbalanced, resulting in microangiopathies and retinal inflammatory injury (Antonetti et al., 1999; Reddy et al., 2024). It can be seen that single-targeted inhibition of vascular disease or inflammatory response has limitations in alleviating diabetic retinopathy, just as there are still 40% of cases of poor response to anti-VEGF antibody drugs (Mounirou et al., 2022), endophthalmitis is its common therapeutic side effect. Consequently, the “anti-inflammation and anti-angiogenesis” dual-target strategy holds greater therapeutic potential.

VEGF-A has become the primary target of DR Therapy due to its strong activity in microvascular diseases. Under the action of hyperglycemia, excessive release of VEGF-A not only causes angiogenesis, but also induces the formation of fragile and leaky vasculature, leading to damage or even loss of vision (Antonetti et al., 1999; Zhang and Ma, 2007). TNF-α, as an important early inflammatory cytokine, also plays an important role in the occurrence and development of DR. The TNF-α-induced inflammatory response will damage the structure and function of the retina, disrupting the integrity of the blood-retinal barrier and the integrity of blood vessels. A meta-analysis of aqueous humor and vitreous cytokines in patients with retinopathy showed significantly higher levels of TNF-α and VEGF-A than in normal subjects (Mason et al., 2022). In this study, we carefully designed a bi-specific nanoantibody Nb-TV that can simultaneously target and bind TNF-α and VEGF-A, and evaluated its anti-inflammatory and anti-angiogenesis activity.

Nanobodies are the smallest antibody fragments, capable of maintaining the selectivity and sensitivity of the corresponding full-length IgG, maximizing the design flexibility. Moreover, the structure of nanobodies is not complicated, and simple tandem modification will not weaken the original targeted binding ability of nanobodies (Wang et al., 2021). The Nb-TV designed by us is constructed by serially connecting Nb-TNFα and Nb-VEGFA end to end through a flexible linker peptide (G4S)_3_, with a molecular weight of only 30.6 kDa. Through simulated molecular docking, Nb-TV is capable of simultaneously targeting and binding TNF-α and VEGF-A. ELISA results reveal that the affinities of Nb-TV for TNF-α and VEGF-A are comparable to or even marginally stronger than those of the parental single-targeted nanobodies, signifying that the activities of the two sites remain unaffected by tandem modification, in accordance with theoretical reports. Meanwhile, Nb-TV effectively reverses the biological effects mediated by TNF-α and VEGF-A. Nb-TV reverses the inflammatory reaction of ARPE-19 induced by TNF-α, as well as the proliferation and migration of HUVECs mediated by VEGF-A. These results suggest that Nb-TV retains the functional activities of the parental antibodies.

Upon activation, immune cells act on RPE cells, inducing the secretion of IL-6, IL-8, MCP-1, *etc*., (Portillo et al., 2017). These factors can both induce inflammatory responses and promote angiogenesis, and are important factors in promoting the progression of DR (Cvitkovic et al., 2020). The RPE increases the production of the above factors through the NF-κB pathway, and TNF-α produced by immune cells is one of the main activating factors of NF-κB. In this study, by detecting p65 nuclear translocation, it was found that TNF-α effectively activates the NF-κB pathway in ARPE-19. The results of Western blot and qPCR showed that the levels of inflammatory markers IL-6, IL-8 and MCP-1 in ARPE-19 were significantly increased. Nb-TV treatment exhibited a significant inhibitory effect on the inflammatory response induced by TNF-α, and its effect was not different from that of Nb-TNFα. However, Nb-VEGFA did not show anti-inflammatory activity. This result indicates that the anti-inflammatory activity of Nb-TV originates from the Nb-TNFα part, which is consistent with our original hypothesis. Wooff et al. found that the NLRP3 inflammasome induces photoreceptor cell necrosis and the initiation of an inflammatory cascade through the Caspase-1 pathway in a photooxidative damage retinopathy model (Wooff et al., 2020), and NLRP3 expression is regulated by the TLR4/NF-κB pathway (Jiang et al., 2024). In this study, the change trend of Caspase-1 level in ARPE-19 was similar. Under the action of TNF-α, its expression was significantly increased, which would promote pyroptosis and further damage the RPE and photoreceptor cells. Nb-TV treatment could significantly reverse the increase in Caspase-1 expression, and this change was consistent with the nuclear translocation of NF-κB p65. Based on this result, Nb-TV targets and binds to TNF-α, blocks the binding of TNF-α and TNFR1, inhibits the activation of the NF-κB pathway, reduces the release of pro-inflammatory cytokines and chemokines, and inhibits the activation of the Caspase-1 pathway, thereby alleviating inflammatory damage and inhibiting pyroptosis, which is of great significance for improving the structure and function of RPE.

In VEGF-A-mediated angiogenesis pathways, ERK1/2-mediated signal transduction in MAPK-ERK pathway is vital for vascular endothelial cell proliferation (Koch et al., 2011), and p38 MAPK activation is essential for initiating endothelial cell migration (Mahabeleshwar et al., 2006). Our results align with this. Under VEGF-A, Erk1/2 was markedly phosphorylated, spurring HUVEC proliferation. Also, p38 MAPK pathway activation led to HUVEC migration. With Nb-TV, Erk1/2 phosphorylation decreased significantly. HUVEC showed no VEGF-A-induced over-proliferation, and p38α had no significant nuclear translocation, effectively curbing cell over-migration. HUVEC is a prevalent *in vitro* model for vascular endothelial cell function research. Under cytokine influence, it can perform tube-forming to mimic blood vessel formation. Paola et al. demonstrated that VEGF-A modulates HUVEC angiogenesis via VEGFR-2/p38 signal transduction (Pignata et al., 2021). In our *in vitro* angiogenesis assays, VEGF-A induced HUVEC migration and subsequent formation of fragments or reticular structures. In the Nb-TV antibody-treated group, HUVEC migration was restrained and vascular structure formation was impeded, suggesting Nb-TV’s inhibitory role in angiogenesis. Vascular lesion is characterized by increased vascular permeability. High glucose markedly elevates iNOS expression in endothelial cells (Shao et al., 2022). iNOS induces NO formation and inflammatory reaction in endothelial cells (Othman et al., 2019), resulting in enhanced endothelial cell permeability. Experimental results revealed minimal iNOS expression under normal conditions. VEGF-A significantly increased iNOS expression in HUVEC cells, signifying increased permeability. Post Nb-TV treatment, iNOS expression was close to that of the control group, suggesting improved permeability. Additionally, Nb-TV’s inhibitory effects on VEGF-A-induced excessive cell proliferation, migration, and permeability increase were similar to those of Nb-VEGFA. Nb-TNFα had no significant effect, implying that Nb-VEGFA function was unaffected by tandem modification.

In both NPDR and PDR, the levels of TNF-α and VEGF-A in the diseased retinas are significantly elevated (Mason et al., 2022; 2025). This is consistent with the results of retinal immunofluorescence in the DR mice of this study.

When Nb-TV was administered to DR mice in the form of eye drops, a remarkable reduction in the levels of both TNF-α and VEGF-A cytokines was observed in the retinas of these mice. In the initial 2-week treatment phase, the single-targeted antibodies primarily exerted their inhibitory effect on the cytokine levels of their respective target molecules. Although, after 4 weeks of treatment, the single-targeted antibody treatments did lead to a partial suppression of the tissue levels of the non-target cytokines, the efficacy was substantially inferior to that of the dual-targeted antibody Nb-TV. The hyperplasia of retinal capillaries is the main cause of visual impairment in DR patients (Li et al., 2016; Zhang et al., 2020). Existing evidence shows that there is a positive correlation between the increased expression of VEGF-A in the hyperglycemic environment of the eye and the number of neovascular vessels (Cui et al., 2020). The results of PAS staining in this study show that Nb-TV can improve the hyperplasia of retinal vessels in DR mice, and the number of neovascular vessels is consistent with the trend of VEGF-A levels detected by immunofluorescence. Meanwhile, Nb-TNFα indirectly inhibits VEGF-A by suppressing the inflammatory response, thus playing a certain role in controlling angiogenesis. The anti-angiogenesis function of Nb-VEGFA is enhanced with the assistance of the anti-inflammatory function of Nb-TNFα, and this synergistic effect is obvious in the Nb-TV group. This further illustrates the close connection between the inflammatory response and angiogenesis, as well as the advantages of the multi - target combined treatment strategy in the treatment of DR.

Our study demonstrates the effectiveness of a dual-targeting strategy “anti-inflammatory and anti-angiogenesis” at the cellular and animal levels; however, several limitations should be acknowledged. First, at the cellular level, the anti-inflammatory and anti-angiogenic activities of the bispecific nanobody were evaluated only in individual cell types. In reality, both inflammation and angiogenesis involve complex multicellular interactions. Therefore, future studies employing co-culture models would improve the physiological relevance and robustness of the findings. Second, compared to commercialized TNF-α nanobodies (whose Kd values are typically at the sub-nM level), such as Ozoralizumab (Kd = 20.2 pM), Nb-TV exhibits nM-level affinity for TNF-α. Although it demonstrates therapeutic efficacy *in vivo* via topical eye drop administration, affinity enhancement strategies such as directed evolution or the introduction of an Fc fragment are still required to reduce the dosage while improving therapeutic outcomes. Third, the cellular experiments in this study primarily focused on changes in the transcription and translation levels of downstream effector proteins following nanobody treatment, without measuring the secretion levels of relevant cytokines. Incorporating cytokine secretion profiling in future studies would provide a more comprehensive understanding of the biological functional effects of this nanobody. Finally, although this study confirmed the therapeutic efficacy of Nb-TV in a mouse model, it did not conduct a comprehensive analysis of the expression of inflammation- and angiogenesis-related factors. Additionally, the significant anatomical and dimensional differences between mouse and human eyes substantially limit the direct clinical translatability of the findings. Future research should further investigate the detailed mechanisms of action, evaluate pharmacokinetics, and validate the mechanisms and efficacy in larger animal models such as rabbits, thereby providing more robust evidence for clinical application.

## 5 Conclusion

In summary, this study, based on the “anti-inflammatory and anti-angiogenesis” dual-target strategy, has successfully constructed a bispecific nanobody, Nb-TV. Nb-TV can simultaneously target TNF-α and VEGF-A, effectively ameliorate vascular hyperplasia and inflammation, thereby exerting a potent therapeutic effect on diabetic retinopathy. Our research not only provides a reliable theoretical and experimental foundation for the application of bispecific nanobodies in the treatment of DR but also uncovers the underlying mechanisms of action. Notably, Nb-TV also holds significant potential application value for other diseases caused by inflammation and abnormal angiogenesis, such as age-related macular degeneration and rheumatoid arthritis. This research paves the way for the development of novel therapeutic approaches for these diseases, highlighting the promising prospects of Nb-TV in the field of biomedicine. We anticipate that further investigations will expand the clinical applications of Nb-TV and contribute to improved patient outcomes.

## Data Availability

The raw data supporting the conclusions of this article will be made available by the authors, without undue reservation.

## References

[B1] Antonetti D. A. Lieth E. Barber A. J. Gardner T. W. (1999). Molecular mechanisms of vascular permeability in diabetic retinopathy. Semin. Ophthalmol. 14, 240–248. 10.3109/08820539909069543 10758225

[B2] Antonetti D. A. Klein R. Gardner T. W. (2012). Diabetic retinopathy. N. Engl. J. Med. 366, 1227–1239. 10.1056/NEJMra1005073 22455417

[B3] Arrigo A. Aragona E. Bandello F. (2022). VEGF-targeting drugs for the treatment of retinal neovascularization in diabetic retinopathy. Ann. Med. 54, 1089–1111. 10.1080/07853890.2022.2064541 35451900 PMC9891228

[B4] Capitão M. Soares R. (2016). Angiogenesis and inflammation crosstalk in diabetic retinopathy. J. Cell Biochem. 117, 2443–2453. 10.1002/jcb.25575 27128219

[B5] Cui K. Liu J. Huang L. Qin B. Yang X. Li L. (2020). Andrographolide attenuates choroidal neovascularization by inhibiting the HIF-1α/VEGF signaling pathway. Biochem. Biophys. Res. Commun. 530, 60–66. 10.1016/j.bbrc.2020.06.130 32828316

[B6] Cvitkovic K. Sesar A. Sesar I. Pusic-Sesar A. Pejic R. Kelava T. (2020). Concentrations of selected cytokines and vascular endothelial growth factor in aqueous humor and serum of diabetic patients. Semin. Ophthalmol. 35, 126–133. 10.1080/08820538.2020.1755320 32310727

[B7] Dong N. Xu B. Shi H. (2018). Long noncoding RNA MALAT1 acts as a competing endogenous RNA to regulate Amadori-glycated albumin-induced MCP-1 expression in retinal microglia by a microRNA-124-dependent mechanism. Inflamm. Res. 67, 913–925. 10.1007/s00011-018-1184-1 30151700

[B8] Feng S. Yu H. Yu Y. Geng Y. Li D. Yang C. (2018). Levels of inflammatory cytokines IL-1β, IL-6, IL-8, IL-17A, and TNF-α in aqueous humour of patients with diabetic retinopathy. J. Diabetes Res. 2018, 8546423. 10.1155/2018/8546423 29850610 PMC5904804

[B9] Hao J. Zhang H. Yu J. Chen X. Yang L. (2019). Methylene blue attenuates diabetic retinopathy by inhibiting NLRP3 inflammasome activation in STZ-induced diabetic rats. Ocul. Immunol. Inflamm. 27, 836–843. 10.1080/09273948.2018.1450516 29608341

[B10] Heloterä H. Kaarniranta K. (2022). A linkage between angiogenesis and inflammation in neovascular age-related macular degeneration. Cells 11, 3453. 10.3390/cells11213453 36359849 PMC9654543

[B11] Jiang K. Zhang F. Chen Y. Li X. Zhao X. Jiang P. (2024). Fosfenopril attenuates inflammatory response in diabetic dry eye models by inhibiting the TLR4/NF-κB/NLRP3 signaling pathway. Invest. Ophthalmol. Vis. Sci. 65, 2. 10.1167/iovs.65.6.2 38829670 PMC11156208

[B12] Kerbel R. S. (2008). Tumor angiogenesis. N. Engl. J. Med. 358, 2039–2049. 10.1056/NEJMra0706596 18463380 PMC4542009

[B13] Koch S. Tugues S. Li X. Gualandi L. Claesson-Welsh L. (2011). Signal transduction by vascular endothelial growth factor receptors. Biochem. J. 437, 169–183. 10.1042/bj20110301 21711246

[B14] Li J. Wang P. Ying J. Chen Z. Yu S. (2016). Curcumin attenuates retinal vascular leakage by inhibiting Calcium/calmodulin-dependent protein kinase II activity in streptozotocin-induced diabetes. Cell Physiol. Biochem. 39, 1196–1208. 10.1159/000447826 27595397

[B15] Lu L. Jiang Y. Jaganathan R. Hao Y. (2018). Current advances in pharmacotherapy and technology for diabetic retinopathy: a systematic review. J. Ophthalmol. 2018, 1694187. 10.1155/2018/1694187 29576875 PMC5822768

[B16] Mahabeleshwar G. H. Feng W. Phillips D. R. Byzova T. V. (2006). Integrin signaling is critical for pathological angiogenesis. J. Exp. Med. 203, 2495–2507. 10.1084/jem.20060807 17030947 PMC2118124

[B17] Mason R. H. Minaker S. A. Lahaie Luna G. Bapat P. Farahvash A. Garg A. (2022). Changes in aqueous and vitreous inflammatory cytokine levels in proliferative diabetic retinopathy: a systematic review and meta-analysis. Eye (Lond). 10.1038/s41433-022-02127-x 35672457

[B18] Mason R. H. Minaker S. A. Lahaie Luna G. Bapat P. Farahvash A. Garg A. (2025). Changes in aqueous and vitreous inflammatory cytokine levels in nonproliferative diabetic retinopathy: systematic review and meta-analysis. Can. J. Ophthalmol. 60, e100–e116. 10.1016/j.jcjo.2024.05.031 39043257

[B19] Mounirou B. A. M. Adam N. D. Yakoura A. K. H. Aminou M. S. M. Liu Y. T. Tan L. Y. (2022). Diabetic retinopathy: an overview of treatments. Indian J. Endocrinol. Metab. 26, 111–118. 10.4103/ijem.ijem_480_21 35873941 PMC9302419

[B20] Othman R. Vaucher E. Couture R. (2019). Bradykinin type 1 receptor - inducible nitric oxide synthase: a new axis implicated in diabetic retinopathy. Front. Pharmacology 10, 300. 10.3389/fphar.2019.00300 30983997 PMC6449803

[B21] Pignata P. Apicella I. Cicatiello V. Puglisi C. Magliacane Trotta S. Sanges R. (2021). Prolyl 3-Hydroxylase 2 is a molecular player of angiogenesis. Int. J. Mol. Sci. 22, 3896. 10.3390/ijms22083896 33918807 PMC8069486

[B22] Portillo J. C. Lopez Corcino Y. Miao Y. Tang J. Sheibani N. Kern T. S. (2017). CD40 in retinal müller cells induces P2X7-Dependent cytokine expression in macrophages/microglia in diabetic mice and development of early experimental diabetic retinopathy. Diabetes 66, 483–493. 10.2337/db16-0051 27474370 PMC5248988

[B23] Reddy S. K. Devi V. Seetharaman A. T. M. Shailaja S. Bhat K. M. R. Gangaraju R. (2024). Cell and molecular targeted therapies for diabetic retinopathy. Front. Endocrinol. (Lausanne) 15, 1416668. 10.3389/fendo.2024.1416668 38948520 PMC11211264

[B24] Robinson R. Glass J. Sharma A. Sharma S. (2022). Generation and characterization of a müller-glial-cell-specific Il6ra knockout mouse to delineate the effects of IL-6 trans-signaling in the retina. Sci. Rep. 12, 17626. 10.1038/s41598-022-22329-3 36271280 PMC9587029

[B25] Rodriguez R. Root H. F. (1948). Capillary fragility and diabetic retinitis; with a note on the use of rutin. N. Engl. J. Med. 238, 391–397. 10.1056/nejm194803182381202 18902950

[B26] Rodríguez M. L. Pérez S. Mena-Mollá S. Desco M. C. Ortega Á L. (2019). Oxidative stress and microvascular alterations in diabetic retinopathy: future therapies. Oxid. Med. Cell Longev. 2019, 4940825. 10.1155/2019/4940825 31814880 PMC6878793

[B27] Semeraro F. Morescalchi F. Cancarini A. Russo A. Rezzola S. Costagliola C. (2019). Diabetic retinopathy, a vascular and inflammatory disease: therapeutic implications. Diabetes Metab. 45, 517–527. 10.1016/j.diabet.2019.04.002 31005756

[B28] Shao J. Ding J. Lu L. Hou W. Wang F. Sun Z. (2022). Propofol protects against high glucose-mediated endothelial injury *via* inhibition of COX2 and iNOS expressions. Acta Biochim. Biophys. Sin. (Shanghai) 54, 548–555. 10.3724/abbs.2022020 35607962 PMC9827823

[B29] Simons M. Gordon E. Claesson-Welsh L. (2016). Mechanisms and regulation of endothelial VEGF receptor signalling. Nat. Rev. Mol. Cell Biol. 17, 611–625. 10.1038/nrm.2016.87 27461391

[B30] Subhi Y. Lykke Sørensen T. (2017). New neovascular age-related macular degeneration is associated with systemic leucocyte activity. Acta Ophthalmol. 95, 472–480. 10.1111/aos.13330 27860298

[B31] Sun S. Ding Z. Yang X. Zhao X. Zhao M. Gao L. (2021). Nanobody: a small antibody with big implications for tumor therapeutic strategy. Int. J. Nanomedicine 16, 2337–2356. 10.2147/ijn.S297631 33790553 PMC7997558

[B32] Teo Z. L. Tham Y. C. Yu M. Chee M. L. Rim T. H. Cheung N. (2021). Global prevalence of diabetic retinopathy and projection of burden through 2045: systematic review and meta-analysis. Ophthalmology 128, 1580–1591. 10.1016/j.ophtha.2021.04.027 33940045

[B33] Tomita Y. Lee D. Tsubota K. Negishi K. Kurihara T. (2021). Updates on the current treatments for diabetic retinopathy and possibility of future oral therapy. J. Clin. Med. 10, 4666. 10.3390/jcm10204666 34682788 PMC8537579

[B34] Wang J. Kang G. Yuan H. Cao X. Huang H. De Marco A. (2021). Research progress and applications of multivalent, multispecific and modified nanobodies for disease treatment. Front. Immunol. 12, 838082. 10.3389/fimmu.2021.838082 35116045 PMC8804282

[B35] Wilkinson C. P. Ferris F. L. Klein R. E. Lee P. P. Agardh C. D. Davis M. (2003). Proposed international clinical diabetic retinopathy and diabetic macular edema disease severity scales. Ophthalmology. 110, 1677–1682. 10.1016/s0161-6420(03)00475-5 13129861

[B36] Wooff Y. Fernando N. Wong J. H. C. Dietrich C. Aggio-Bruce R. Chu-Tan J. A. (2020). Caspase-1-dependent inflammasomes mediate photoreceptor cell death in photo-oxidative damage-induced retinal degeneration. Sci. Rep. 10, 2263. 10.1038/s41598-020-58849-z 32041990 PMC7010818

[B37] Yu H. Lin L. Zhang Z. Zhang H. Hu H. (2020). Targeting NF-κB pathway for the therapy of diseases: mechanism and clinical study. Signal Transduct. Target Ther. 5, 209. 10.1038/s41392-020-00312-6 32958760 PMC7506548

[B38] Zhang S. X. Ma J. X. (2007). Ocular neovascularization: implication of endogenous angiogenic inhibitors and potential therapy. Prog. Retin Eye Res. 26, 1–37. 10.1016/j.preteyeres.2006.09.002 17074526

[B39] Zhang J. Zhang M. Zhao H. Xu X. (2020). Identification of proliferative diabetic retinopathy-associated genes on the protein-protein interaction network by using heat diffusion algorithm. Biochim. Biophys. Acta Mol. Basis Dis. 1866, 165794. 10.1016/j.bbadis.2020.165794 32278010

